# Human Sperm Cryopreservation: Update on Techniques, Effect on DNA Integrity, and Implications for ART

**DOI:** 10.1155/2012/854837

**Published:** 2011-12-13

**Authors:** Marlea Di Santo, Nicoletta Tarozzi, Marco Nadalini, Andrea Borini

**Affiliations:** Tecnobios Procreazione, Centre for Reproductive Health, Via Dante 15, 40125 Bologna, Italy

## Abstract

Cryopreservation of human spermatozoa—introduced in the 1960's—has been recognized as an efficient procedure for management of male fertility before therapy for malignant diseases, vasectomy or surgical infertility treatments, to store donor and partner spermatozoa before assisted reproduction treatments and to ensure the recovery of a small number of spermatozoa in severe male factor infertility. Despite the usefulness of it, cryopreservation may lead to deleterious changes of sperm structure and function: while the effects of cryopreservation on cells are well documented, to date there is no agreement in the literature on whether or not cryopreservation affects sperm chromatin integrity or on the use of a unique and functional protocol for the freezing-thawing procedure. Therefore, sperm cryopreservation is an important component of fertility management and much of its successful application seems to affect the reproductive outcome of assisted reproduction technologies (ART): appropriate use of cryoprotectants before and sperm selection technologies after cryopreservation seem to have the greatest impact on preventing DNA fragmentation, thus improving sperm cryosurvival rates.

## 1. Introduction

The procedure that makes it possible to stabilize the cells at cryogenic temperatures is called cryopreservation, also known as an applied aspect of cryobiology or the study of life at low temperatures. Many advances in the cryopreservation technology have led to the development of methods that allow for low-temperature maintenance of a variety of cell types including male and female gametes, small multicellular organisms, and even more complex organisms such as embryos. Cryopreservation of human spermatozoa—introduced in the 1960's [1]—has overcome many space and time limitations and now forms integral part of assisted reproduction technologies (ARTs). 

This technique becomes particularly important in cases of preservation of male fertility before radiotherapy or chemotherapy [2] which may lead to testicular failure or ejaculatory dysfunction. In fact, semen cryostorage seems to be the only proven method that may offer these couples a chance of having children in the future: cancer therapy could in fact lead to damage, resulting in subfertility or sterility due to gonad removal or permanent damage to germ cells caused by adjuvant therapy. In particular, the risk associated to therapy depends on several factors: the age of the patient at the time of treatment, the dose, site, and type of treatment [3]. Also some nonmalignant diseases, such as diabetes and autoimmune disorders, may lead to testicular damage. Cryopreservation is advisable also in these conditions [4]. In countries in which heterologous fertilization is allowed by law and in donor insemination programmes cryopreservation is necessary to have enough time to screen donors for infectious agents, such as the human immunodeficiency (HIV) and hepatitis B viruses, before the cryopreserved semen is used for clinical purposes [5]. In azoospermic patients, who have undergone testicular sperm extraction or percutaneous epididymal sperm aspiration, sperm cryostorage is also used to avoid repeated biopsies or aspirations [6]. Furthermore, cryopreservation is routinely performed in patients who—having to start an assisted reproduction treatment—decide to preemptively freeze the semen sample to avoid inconveniences due to failed ejaculation often associated with “semen collection stress,” certain emotional states, or other commitments at the time of oocyte retrieval [7]. Finally, male gamete freezing is largely recommended to preserve fertility in those subjects who—for one reason or another—are exposed to potentially toxic agents which may interfere with gametogenesis [7]. 

## 2. Techniques for Cryopreservation

There are two main conventional freezing techniques used in sperm cryopreservation: slow freezing and rapid freezing 

### 2.1. Slow Freezing

The slow freezing technique proposed by Behrman and Sawada [8] consists of progressive sperm cooling over a period of 2–4 h in two or three steps, either manually or automatically using a semiprogrammable freezer.

The manual method is performed by simultaneously decreasing the temperature of the semen while adding a cryoprotectant in a stepwise manner and after plunging the samples into liquid nitrogen [9]. It has been shown that the optimal initial cooling rate of the specimen from room temperature to 5°C is 0.5–1°C/min [10]. The sample is then frozen from 5°C to −80°C at a rate of 1–10°C/min. The specimen is then plunged into liquid nitrogen at −196°C [11]. 

In spite of reports showing successful sperm freezing with manual techniques, the reproducibility of this procedure could pose some problems. For this reason, programmable freezers have been investigated [12]. The freezers use a plate to hold the straws; these are cooled by liquid nitrogen held in a storage tank under the plate. Liquid nitrogen is poured into the tank, and the machine, once programmed, uses the software data logging to obtain cooling from 20°C to −80°C at rate of 1.5°C/min and then at 6°C/min; at completion of the freezing the straws are removed and stored into liquid nitrogen at −196°C. This takes about 40 min [12]. Programmes are simple to use and allow for a cooling combination which does not require continuous operator intervention and have been used to increase the reproducibility of the freezing operations [12].

Some authors argue that conventional slow freezing, either *manual* or *automated*, causes extensive chemical-physical damage to the sperm probably because of ice crystallization [13].

### 2.2. Rapid Freezing

Rapid freezing was first proposed by Sherman [14]. This technique requires direct contact between the straws and the nitrogen vapours for 8–10 min and immersion in liquid nitrogen at −196°C. Inside nitrogen vapours there is a thermal gradient, as a function of the distance and the volume of the liquid below. The sample is initially mixed in dropwise manner with equal volume of cold cryoprotectant; the mixture is loaded into the straws and left to incubate at 4°C for 10 minutes. The straws are then placed at a distance of 15–20 cm above the level of liquid nitrogen (−80°C) for 15 min; after this stage, the straws are immersed in liquid nitrogen. During cooling it is preferable to place the straws in horizontal position to minimize the heat difference between the two ends. This technique has some drawbacks among which; for example, low reproducibility, indeed, the temperature drop curve cannot be controlled, and the freezing temperatures may vary from −70, −80, and −99°C [7].

### 2.3. Cryopreservation of Small Numbers of Spermatozoa

The conventional methods of sperm cryopreservation described above are not ideal to cryopreserve small numbers of cells, such as epididymal and testicular spermatozoa. Efficient cryopreservation of surgically retrieved spermatozoa, as mentioned earlier in this chapter, reduces the number of surgical interventions and avoids the logistic problem associated with coordinating the women's oocyte retrieval and also the risk of no sperm being found on the day of oocyte retrieval [15]. Thus, novel cryopreservation approaches have been designed to cryopreserve limited numbers of motile sperms in a very small volume (*Table 1*). Both biological and nonbiological carriers have been tried for the cryopreservation of low numbers of spermatozoa, although, to date, no prospective randomised trials have been conducted to demonstrate that any single carrier is superior to the others [15]. Furthermore, to date there is a limited use of these technologies in the majority of IVF programs. This suggests that novel cryopreservation technology, designed to handle small sperm numbers and needs to be further explored [15]. Whatever the method used, to obtain good results it is essential to correctly perform all the steps before and after cryopreservation: the choice of more suitable cryoprotectants and the thawing procedure are particularly important.

### 2.4. Cryoprotectants

Cryoprotectants are low-molecular-weight and highly permeable chemicals used to protect spermatozoa from freeze damage by ice crystallization. There are four main well-known cryoprotectants: glycerol, ethylene glycol, dimethyl sulphoxide, and 1,2-propanediol. Cryoprotectants act by decreasing the freezing point of a substance, reducing the amount of salts and solutes present in the liquid phase of the sample and by decreasing ice formation within the spermatozoa [16]. Usually the cryoprotectants are added in an equal volume of semen in a dropwise manner, gently mixed at room temperature, and then placed at 37°C for 10–15 minutes to allow for proper equilibration between the cells and the medium [7]. It is necessary that the medium interacts with the cells. Indeed, the effectiveness of cryoprotecting substances is also a function of the time of interaction between the cryoprotectants and the cells [7]. Glycerol is the permeating cryoprotectant most widely used for human sperm acting on: the membrane structure, permeability and stability of the lipid bilayer, the association of surface proteins and the cellular metabolism. Its employment gives an unfavorable outcome on membrane and acrosome structure, although allowing the freezing of poor quality sperm [7]. Sherman's [14] studies showed that the use of glycerol may cause few alterations such as: presence of an undulating membrane, alteration in acrosomal internal membrane, nucleus inhomogeneity and disorganization in mitochondrial crests. Following this observations other protective substances were proposed such as the dimethyl sulfoxide (DMSO), which has deleterious effects on human sperm when used at 4°C, and the 1,2-propanediol slightly used in sperm cryopreservation [7].

### 2.5. Thawing Procedure

The thawing procedure is an equally important step: the cell must be allowed to recover its normal biological activities trying to avoid abrupt thermal changes as far as possible. Generally speaking, the cryopreservation protocols use a thawing temperature of 37°C; even if higher thawing temperatures allow for more rapid heating, they are not used because of the risks associated to cell damage. 

At the present time, several thawing techniques are used, among which are

- thawing at room temperature for 10 min and subsequent thermostat pass at 37°C for another 10 min,
- thawing in a thermostat and water-bath at 37°C for 10 min,
- thawing at room temperature for 15 min.

Once the semen is thawed, it is separated from the cryopreservation medium by washing in culture medium and centrifuging [7].

## 3. Detrimental Effects of Cryopreservation on Sperm Integrity

Compared with other cell types, spermatozoa seem to be less sensitive to cryopreservation damage because of the high fluidity of the membrane and the low water content (about 50%). Despite this, cryopreservation may lead to deleterious changes of sperm structure and function [17]. It was largely reported that several damaging processes could occur during freezing-thawing of human spermatozoa, such as thermal shock with formation of intracellular and extracellular ice crystals, cellular dehydration, and osmotic shock [18].

The primary cause of cellular damage during cryopreservation is the formation of intracellular or extracellular ice crystals. During the freezing process, the cooling rate plays an important role in determining the extent of cryoinjury to the spermatozoa [9].

A rapid cooling rate causes severe intracellular ice formation, since the efflux of water across the membrane is impaired, thus, inducing supercooling. 

Ice crystals formed breach the membranes and affect the organelle function. This condition leads to impaired cell survival. On the other hand, a too slow cooling rate determines the efflux of water from the internal to the external environment, increasing the concentration of solutes and the osmotic pressure. This condition leads to cell volume changes associated with the movement of water, dehydration, and toxicity damage due to high solute concentration [9]. Cryoinjury is not limited to the freezing process but may also occur during the thawing process as the ice melts or recrystallizes [9]. The phenomenon of recrystallization of both intracellular and extracellular ice, in frozen samples, occurs as smaller ice crystals with a rate of recrystallization that increases with increasing temperature [13].

It has largely been reported that chilling injury can modify the structure and integrity of plasma membranes [19, 20] mainly composed by phospholipids and cholesterol [21]. 

Even though high concentrations of cholesterol and polyunsaturated fatty acids give more fluidity to the membrane at lower temperature [22], during cryopreservation the cooling process causes phase transition of membrane lipids and impairs membrane protein function. In particular, the outer layer of the spermatozoal plasma membrane consists of a glycocalyx, a carbohydrate-rich zone that mainly contains oligosaccharide chains that bind to the integral protein of the plasma membrane (glycoproteins) or lipids (glycolipids) [23]. Generally, cryopreservation may have a detrimental effect changing the carbohydrate composition of the glycocalyx, thus impairing the function of membrane proteins which are responsible for ion transport and metabolism and affecting the fertilizing ability [24]: the glycocalyx is involved in some physiological functions such as immune protection of the female genital tract [25], acrosomal reaction [26], and early gamete interaction.

The plasma and mitochondrial membranes have the same susceptibility to cryopreservation [27]. Mitochondria are placed along the midpiece between the plasma membrane and the nine fibrous columns, to form a coating that provides energy necessary for sperm motility [27]. The greatest amount of energy is provided by molecules of ATP synthesized either by glycolysis in the cytoplasm [28] or through oxidative phosphorylation (oxphos) in the mitochondria [10]. 

The ATP generated by oxphos in the inner mitochondrial membrane is transferred to the microtubules, to drive motility [29]. Therefore, an impairment of mitochondrial activity may explain the reduction in motility [27]. 

An alteration in mitochondrial membrane fluidity may also lead to an alteration in mitochondrial membrane potential and release of ROS [9]. The peroxidative damage induced by increased concentration of ROS is associated with damage to the sperm plasma membrane and impairment of the axonemal structure [30]. In addition, cryopreservation has been shown to diminish the antioxidant activity of the spermatozoa making them more susceptible to ROS damage [31]. High concentration of ROS and fall of antioxidant enzymes lead to cell apoptosis [32]. In this context the apoptosis cascade is mediated by activation of the BCL2 family proteins: there is a permeabilization of the outer mitochondrial membrane through the Bax and BAK proteins and the release of cytochrome [33]. In turn, caspase 9 is activated along with APAF-1 to form an apoptosome [34]. The release of apoptosis-inducing factors from the mitochondria leads to DNA fragmentation [35]. Several studies examined the role of *in vitro* antioxidant supplementation in protecting the sperm DNA from oxidative damage. For example, when added to the seminal fluid during cryopreservation, genistein [36], resveratrol [37], and ascorbic acid [37] seem to reduce DNA damage; on the contrary, vitamin E [38], ascorbate, and catalase [39] seem to improve motility and reduce ROS levels, though they do not improve spermatozoal viability and do not reduce DNA damage. In any case, the number of these studies and the number of patients they include is still too limited to draw any conclusions on the efficacy of antioxidant supplementation in protecting DNA from freezing-induced damage.

### 3.1. Cryopreservation and DNA Damage

While the effects of cryopreservation on the fertilization capacity, motility, morphology, and viability of spermatozoa are well documented, still open is the question of the possible alteration of sperm DNA integrity after freezing-thawing procedures. There is no agreement in the literature neither on whether cryopreservation induces DNA damage nor on the amount of damage. In some studies, authors have reported significant alterations of sperm DNA integrity after cryopreservation/thawing [6, 40], whereas other studies have expressed a different opinion [41, 42]. This controversy between one study and the other could first of all be explained by the fact that the findings do not refer to a considerable number of samples and is also due to the use of (1) different freezing procedures, (2) different tests to evaluate the DNA integrity, and (3) different semen preparation techniques before cryopreservation (i.e., swimup or density gradient centrifugation). For example, Donnelly and colleagues [6] investigated pre-cryopreservation and postcryopreservation DNA integrity of both semen and prepared sperm samples (density gradient centrifugation or direct swimup) in 50 men. They reported that freezing sperm in seminal plasma improves postthaw DNA integrity: sperm-frozen unprepared in seminal fluid seems to be more resistant to freezing damage than frozen prepared sperm; further improvement can be achieved by preparing sperm and freezing after readdition of seminal plasma. This may be due to the presence of abundant antioxidants in seminal plasma. Concerning the variability linked to different freezing procedures, in the study of Petym and colleagues [43], the authors evaluated cryodamage on sperm chromatin comparing two different procedures: liquid nitrogen vapour versus computerized program freezer. They analyzed 50 semen samples using acridine orange and concluded that DNA damage was significantly higher following freezing with liquid nitrogen. 

From a detailed analysis of the references currently available in the literature, it was found that there are basically three different lines of thought about the question: “*Does the freezing-thawing procedure induce sperm DNA damage?*”. 

According to several authors the answer is “*YES” *(Table 2(a)). Spano and his group [44] reported that overall sperm quality deteriorates after freezing-thawing, including sperm DNA integrity assessed by SCSA in 19 samples. These findings have been confirmed in a study by De Paula and colleagues [40] on 77 patients, where the authors have evaluated the degree of sperm DNA fragmentation by TUNEL assay before and after cryopreservation: the authors stressed that the freezing-thawing procedure negatively affects DNA integrity. Furthermore, from the data published in the literature, it is also clear that among the authors who argue that cryopreservation induces sperm DNA damage there is sometimes no agreement on the mechanism which actually induces that damage. For example, in spite of the fact that ROS was widely reported to play an important role in the pathophysiology of damage to human spermatozoa, including DNA fragmentation, Zribi and his group [45] stated that there is no relationship between DNA fragmentation and DNA oxidation. They suggested that cryopreservation induces sperm DNA fragmentation through other pathways beside oxidative stress, such as defects in DNA repairing enzymes or enhancement of defects already present in sperm cells. This hypothesis is controverted by Thomson and colleagues [46]: despite the use of the same technique to assess DNA oxidation, fluorescent assay for the detection of 8 oxoguanine, they reported that human sperm DNA fragmentation is associated with an increase in oxidative stress during cryopreservation, rather than the activation of caspase and apoptosis.

About the question “*Does the freezing-thawing procedure induce sperm DNA damage?*” some authors follow another line of thought and answer “*YES, but with some conditions*” (Table 2(b)). They support the hypothesis of less susceptibility to freezing damage in the spermatozoa of fertile men, classified using WHO criteria, than those of infertile men. In the study of Donnelly and colleagues [6], semen samples were obtained from 17 fertile and 40 infertile men, and sperm integrity was assessed before and after cryopreservation using the Comet assay. The authors showed that semen from fertile men appears to be more resistant to freezing damage than sample from infertile men; moreover, in fertile man, there was no significant decrease in DNA integrity after cryopreservation. These results support the observation that spermatozoa from infertile men have a greater incidence of irregular chromatin organization and show significantly decreased resistance to thermal denaturation compared with spermatozoa from fertile men [47, 48]. In fact, as a consequence of reduced protamination, poor-quality spermatozoa often contain partially decondensed chromatin that generates functional immaturity [6]. Chromatin condensation is fundamental for spermatozoa since spermatogenesis results in the discarding of cytoplasm, leaving the spermatozoa incapable of undertaking DNA repair. According to this hypothesis, Kalthur and colleagues [49], evaluating sperm morphology and sperm DNA damage before and after cryopreservation, reported that the susceptibility of morphologically abnormal sperm to DNA damage during the freezing process is significantly higher than that of sperm with normal morphology. They hypothesised that sperm with head abnormalities may have altered membrane physical properties and thereby have altered tolerance to cold stress. However, there are no studies conducted to assess whether or not a morphologically abnormal sperm can retain its chromatin integrity during cryopreservation.

In opposition to these two answers to the above question, there is a third line of thought: some authors say: “*NO, the freezing-thawing procedure does not compromise sperm DNA integrity*” (Table 2(c)). For example, Duru and his group [41] evaluated the effects of cryopreservation on DNA fragmentation and membrane integrity in 21 patients using the TUNEL assay and annexin V. Their results indicated that cryopreservation altered plasma membrane symmetry and was associated with translocation of phosphatidylserine, while DNA integrity was maintained. In addition, Isachenko and colleagues [42], comparing the effects of slow freezing and vitrification on sperm DNA integrity in the absence of cryoprotectant, found that the integrity of DNA is unaffected by cryopreservation. The lack of effects of cryopreservation on sperm DNA has also been confirmed by data of Paasch and colleagues [50]. They demonstrated that cryopreservation was significantly associated with disruption of the mitochondrial membrane potential, as well as activation of caspase 3, 8, and 9, but no significant changes were observed in DNA fragmentation, as assessed by the TUNEL assay in 84 samples. 

Even if the opinions on the issue of “*cryopreservation and DNA damage*” are still highly controversial, the evaluation of the impact of cryopreservation on sperm chromatin is of extreme importance. Likewise, sperm DNA integrity is an important factor for the success of ART [51–53]. 

## 4. Cryopreservation and Reproductive Outcome

Cryopreservation is widely known to raise impaired sperm motility and decrease fertilization rate through detrimental effects on membranes, acrosomal structure, and acrosin activity [54]. The freezing-thawing procedure of human spermatozoa may also be detrimental to the chromatin structure [55], leading to a potential risk of decondensation of the sperm nucleus after injection into the oocyte, thus, reducing fertilization rate [56]. However, a cumulative effect of cryopreservation on sperm fertilization capacity is not definitely established. Considering the decrease in sperm fertilization power induced by cryopreservation, it can be easily understood that intrauterine insemination and conventional *in vitro* fertilization (IVF) with frozen-thawed spermatozoa result in lower pregnancy rates compared with insemination with fresh sperm [1]: this is the reason why cryopreservation of semen samples before intrauterine insemination or conventional IVF is not recommended. 

The considerations are different for intracytoplasmic sperm injection (ICSI), because this procedure requires only a small number of motile spermatozoa for successful fertilization. Therefore, the current question is whether using fresh rather than cryopreserved sperm cells has the same effect on reproductive outcome in ICSI. To date, only a few large-scale studies on ICSI reproductive outcome comparing fresh and frozen-thawed human ejaculated, testicular, or epididymal spermatozoa have been reported in the literature, and the results seem to differ between the authors also depending on the origin of the employed spermatozoa.

### 4.1. Testicular Spermatozoa

Friedler and colleagues [57] reported no statistically significant differences in all parameters examined (fertilization rate, cleavage rate, embryo quality, implantation rate, clinical pregnancy rate, and ongoing pregnancy rate) between ICSI cycles with fresh or cryopreserved testicular spermatozoa from the same patients, comparing all ICSI cycles performed with fresh (25 cycles) and thawed (14 cycles) testicular spermatozoa, respectively. This hypothesis is supported by the study of some authors: Ben Rhouma and colleagues [58] performed a total of 60 ICSI cycles with fresh (32 cycles) and thawed (28 cycles) testicular spermatozoa; Habermann and colleagues [59] performed a total of 46 ICSI cycles with fresh (12 cycles) and thawed (34 cycles) testicular spermatozoa; Huang and colleagues [60] performed a total of 22 ICSI cycles with fresh (14 cycles) and thawed (8 cycles) testicular spermatozoa. All the authors reported the same results: the reproductive outcome of ICSI with frozen-thawed testicular spermatozoa is comparable with the reproductive outcome of ICSI obtained with fresh testicular spermatozoa. In contrast, De Croo and colleagues [61] stated that fertilization, implantation, and live-birth rates per embryo transfer are significantly lower after ICSI with frozen-thawed (35 cycles) than those with fresh (65 cycles) testicular spermatozoa.

### 4.2. Epididymal Spermatozoa

On the other hand, some groups compared the results of intracytoplasmic sperm injection (ICSI) with fresh and frozen-thawed epididymal spermatozoa. For example, Tournaye and colleagues [62] reported that the clinical pregnancy rate in ICSI cycles was comparable between fresh (157 cycles) and frozen-thawed (118 cycles) epididymal spermatozoa. Sukcharoen and colleagues [63] performed a total of 53 ICSI cycles with fresh (40 cycles) and thawed (13 cycles) epididymal spermatozoa and came to the same conclusion; also Cayan and colleagues [64] supported the same opinion. In opposition Shibahara and colleagues [65] stated that there was a significant difference in all reproductive parameters examined between ICSI cycles with fresh or cryopreserved epididymal spermatozoa, comparing ICSI cycles performed with fresh (5 cycles) and thawed (13 cycles) epididymal spermatozoa.

### 4.3. Ejaculated Spermatozoa

The majority of studies on cryopreservation and ICSI reproductive outcome were conducted using spermatozoa of testicular or epididymal origin. Only two major groups reported data on fertilization and pregnancy rates after ICSI comparing fresh and frozen-thawed human ejaculated spermatozoa. First, Kucznynski and colleagues [66] compared the reproductive outcome of 118 ICSI cycles using fresh spermatozoa and 122 ICSI cycles using frozen-thawed spermatozoa, all from oligoasthenoteratozoospermic patients. The authors did not report of any statistically significant differences in fertilization rate between the two groups of patients. Moreover, these data show that values of ongoing pregnancies are significantly higher in ICSI patients when human sperm samples are cryopreserved. According to Ragni and his group [67], this suggests that properly performed cryopreservation selectively affects defective rather than normal spermatozoa [44]. This observation seems to indicate that cryopreservation before ICSI might be helpful to eliminate senescent or deficient spermatozoa, thus, improving reproductive outcome [62]. Borges and his group [68] also investigated sperm cryopreservation effects on ICSI outcome. The author compared 61 and 79 ICSI cycles performed with cryopreserved and fresh ejaculated spermatozoa and, in particular, examined the reproductive outcome obtained using semen samples with decreased and with normal motility. Results demonstrated that (1) using semen with normal motility the reproductive outcome obtained using fresh or frozen-thawed spermatozoa is the same; (2) in semen with decreased motility the fertilization rate with fresh sperm was higher than that with the cryopreserved one, but no differences were detected in implantation and pregnancy. This finding supports the hypothesis that the freezing-thawing procedure causes more damage in patients with alterations in semen quality than that in patients with normal semen. However, once the oocyte is fertilized, implantation and pregnancy rates are similar in patients with or without sperm anomalies. 

## 5. Conclusion

Today, sperm cryopreservation is widely used to store donor and partner spermatozoa before assisted reproduction treatments, to preserve spermatozoa before therapy for malignant diseases, vasectomy, or surgical infertility treatments and to ensure the recovery of a small number of spermatozoa in severe male factor infertility. Therefore, sperm cryopreservation is an important component of fertility management, and much of its successful application seems to affect the reproductive outcome of ART. 

While the effects of cryopreservation on cells are well documented, to date there is no agreement in the literature on whether or not cryopreservation affects sperm chromatin integrity or on the use of a unique and functional protocol for the freezing-thawing procedure. This suggests that, to date, it would be useful to perform a multicenter study with large numbers of semen specimens which could be processed using unique freezing protocols. Moreover, though further investigations are needed to fully understand the real influence of cryopreservation on sperm DNA integrity and the impact of the use of cryopreserved spermatozoa on the reproductive outcome, technical measures should be applied to provide maximum protection to the male gametes: appropriate use of cryoprotectants before and sperm selection technologies after cryopreservation seems to have the greatest impact on preventing DNA fragmentation, thus improving sperm cryosurvival rates. 

## Figures and Tables

**Table 1:** Approaches to cryopreserve limited number of spermatozoa.

Cryopreservation techniques	Authors	Principle	Main advantages	Main disadvantages
Empty zona pellucida	Borini et al. [69]	Storage of individual spermatozoa in animal or human empty zona pellucida.	Avoid waste of time in screening to locate motile sperm; cryoprotectants can be added and removed without loss of spermatozoa sequestered in the zona	Risk of biological contamination
	Cohen et al. [70]			
	Walmsley et al. [71]			
	Montag et al. [72]			
	Hsieh et al. [73]			
	Liu et al. [74]			
	Levi-Setti et al. [75]			
	Cesana et al. [76]			
	Hassa et al. [77]			

Microdroplets	Gil-Salom et al. [78]	Storage of droplets of sperm/cryoprotectants mixture on the surface of dry ice and directly plunged into liquid nitrogen	Avoid sperm loss through adherence to the vessel	Risk of cross-contamination; shape and size of dishes make difficult to handle and store in conventional freezers and liquid nitrogen tanks
	Sereni et al. [79]			
	Quintans et al. [80]			
	Bouamama et al. [81]			

ICSI pipette	Gvakharia et al. [82]	Storage of spermatozoa in ICSI pipettes	Sterile, simple, and convenient system	Not practical for long-term storage; fragility of ICSI pipettes; risk of cross-contamination
	Sohn et al. [83]			

*Volvox globator *spheres	Just et al. [84]	Storage of sperm into spheres of *Volvox globator *	Significant postthaw recovery of motile sperm	Exposure to genetic material from the algae; constant source of algae

Alginate beads	Herrler et al. [85]	Microencapsulation in alginate beads	Inert nature of alginate beads	Decrease sperm motility with encapsulation

Cryoloop	Nawroth et al. [86]	Individual spermatozoa deposited directly on cryoprotectant film covering the nylon loop and immersed in liquid nitrogen	Excellent vessel for vitrification; no additional preparation	Open system: risk of cross-contamination
	Schuster et al. [87]			
	Isachenko et al. [42]			
	Isachenko et al. [42]			
	Desai et al. [88]			
	Desai et al. [89]			

Agarose microspheres	Isaev et al. [90]	Storage of sperm loaded in agarose microspheres	Nonbiological carrier	Clinical value of this approach not evaluated

Straws	Desai et al. [91]	Sperm/cryoprotectants loaded into the ministraw	Sterile, simple, and convenient system	Not ideal for severely impaired specimens; sperm loss due to adherence to the vessel
	Isachenko et al. [92]			
	Koscinski et al. [93]			

**Table:** (a)

Authors	Test to evaluate DNA integrity	Number of samples	Cryopreservation method	“Does the freezing-thawing procedure induce sperm DNA damage?”
Hamamah et al. [94]	Acridine orange staining and Feulgen-DNA quantitative microspectrophotometry	10	Unspecified	Yes

Spanò et al. [44]	SCSA + Acridine orange staining	19	Equilibration at 37°C, freezing in liquid nitrogen vapour at −80°C and then storage in liquid nitrogen at –196°C	Yes

Hammadeh et al. [95]	Acridine orange staining	59	Computerized slow-stage freezer + static liquid nitrogen vapour	Yes

Donnelly et al. [6]	COMET assay	40	Equilibration at 37°C, freezing in liquid nitrogen vapour at −80°C and then storage in liquid nitrogen at –196°C	Yes

Gandini et al. [96]	Acridine orange staining	19	Equilibration at 37°C, freezing in liquid nitrogen vapour at −80°C and then storage in liquid nitrogen at –196°C	Yes

de Paula et al. [40]	TUNEL assay	77: (i) 30 normozoospermic (ii) 47 oligozoospermic	Use of freezer at –20°C, freezing in liquid nitrogen vapour, then storage in liquid nitrogen –196°C	Yes

Petyim and Choavaratana [43]	Acridine orange staining	50	Freezing with liquid nitrogen vapour + computerized program freezer	Yes

Nagamwuttiwong and Kunathikom [97]	Acridine orange staining	20	Freezing with liquid nitrogen vapour	Yes

Dejarkom and Kunathikom [98]	Acridine orange staining	20	Computerized controlled rate freezing	Yes

Thomson et al. [46]	TUNEL assay	60	Use of programmable freezer	Yes

Thomson et al. [46]	TUNEL assay	320	Sample frozen with and without cryoprotectant by slow-controlled-rate method using a programmable freezer	Yes

Zribi et al. [45]	TUNEL assay	15	Equilibration at 37°C, freezing in liquid nitrogen vapour at −80°C, then storage in liquid nitrogen at –196°C	Yes

**Table:** (b)

Authors	Test to evaluate DNA integrity	Number of samples	Cryopreservation technique	“Does the freezing-thawing procedure induce sperm DNA damage?”
Donnelly et al. [6]	COMET assay	57: (i) 17 fertile (ii) 40 infertile	Equilibration at 37°C, freezing in liquid nitrogen vapour at −80°C, then storage in liquid nitrogen at –196°C	Yes, but semen from fertile men appears to be more resistant to freezing damage

Kalthur et al. [49]	COMET assay + Acridine orange staining	44	Equilibration at 37°C, static cooling at 4°C, cooling vapour phase, then storage in liquid nitrogen at –196°C	Yes, but morphologically abnormal sperms seems to be less resistant to freezing damage

Ahmad et al. [99]	COMET assay	196: (i) 30 normospermic (ii) 166 infertile	Freezing with static-phase vapour cooling procedure	Yes, but the sperm DNA integrity of frozen samples of fertile men is higher

**Table:** (c)

Authors	Test to evaluate the DNA integrity	Number of samples	Cryopreservation technique	“Does the freezing-thawing procedure induce sperm DNA damage?”
Høst et al. [100]	Immunoperoxidase detection of digoxigenin-labelled genomic DNA	53: (i) 20 fertile (ii) 33 infertile	Conventional cryopreservation	No

Steele et al. [101]	COMET assay	21: (i) 9 control (ii) 12 with obstructive azoospermia	Freezing in liquid nitrogen vapour	No

Duru et al. [41]	TUNEL assay + annexin V	21	Equilibration at 37°C, freezing in liquid nitrogen vapour at −80°C, then storage in liquid nitrogen at –196°C	No

Isachenko et al. [42]	COMET assay	18	Programmable slow freezing + vitrification	No

Paasch et al. [50]	TUNEL assay + flow cytometric kit for apoptosis	84	Freezing at –20°C, freezing in liquid nitrogen vapor at –100°C, then storage in liquid nitrogen at –196°C	No

## References

[1] Sherman JK (1973). Synopsis of the use of frozen human semen since 1964: state of the art of human semen banking. *Fertility and Sterility*.

[2] Sanger WG, Olson JH, Sherman JK (1992). Semen cryobanking for men with cancer—Criteria change. *Fertility and Sterility*.

[3] Jensen JR, Morbeck DE, Coddington III CC (2011). Fertility preservation. *Mayo Clinic Proceedings*.

[4] Anger JT, Gilbert BR, Goldstein M (2003). Cryopreservation of sperm: indications, methods and results. *Journal of Urology*.

[5] Morris GJ, Acton E, Avery S (1999). A novel approach to sperm cryopreservation. *Human Reproduction*.

[6] Donnelly ET, McClure N, Lewis SEM (2001). Cryopreservation of human semen and prepared sperm: effects on motility parameters and DNA integrity. *Fertility and Sterility*.

[7] Fabbri R, Ciotti P, Di Tommaso B (2004). Tecniche di crioconservazione riproduttiva. *Rivista Italiana di Ostetricia e Ginecologia*.

[8] Behrman SJ, Sawada Y (1966). Heterologous and homologous inseminations with human semen frozen and stored in a liquid-nitrogen refrigerator. *Fertility and Sterility*.

[9] Said TM, Gaglani A, Agarwal A (2010). Implication of apoptosis in sperm cryoinjury. *Reproductive BioMedicine Online*.

[10] Mahadevan M, Trounson AO (1984). Effect of cooling, freezing and thawing rates and storage conditions on preservation of human spermatozoa. *Andrologia*.

[11] Thachil JV, Jewett MAS (1981). Preservation techniques for human semen. *Fertility and Sterility*.

[12] Holt WV (2000). Basic aspects of frozen storage of semen. *Animal Reproduction Science*.

[13] Mazur P, Rall WF, Rigopoulos N (1981). Relative contributions of the fraction of unfrozen water and of salt concentration to the survival of slowly frozen human erythrocytes. *Biophysical Journal*.

[14] Sherman J, Keel B, Webster BW (1990). Cryopreservation of human semen. *Handbook of the Laboratory Diagnosis and Treatment of Infertility*.

[15] Abdelhafez F, Bedaiwy M, El-Nashar SA, Sabanegh E, Desai N (2009). Techniques for cryopreservation of individual or small numbers of human spermatozoa: a systematic review. *Human Reproduction Update*.

[16] Royere D, Barthelemy C, Hamamah S, Lansac J (1996). Cryopreservation of spermatozoa: a 1996 review. *Human Reproduction Update*.

[17] Watson PF (2000). The causes of reduced fertility with cryopreserved semen. *Animal Reproduction Science*.

[18] Stanic P, Tandara M, Sonicki Z, Simunic V, Radakovic B, Suchanek E (2000). Comparison of protective media and freezing techniques for cryopreservation of human semen. *European Journal of Obstetrics Gynecology and Reproductive Biology*.

[19] Arav A, Zeron Y, Leslie SB, Behboodi E, Anderson GB, Crowe JH (1996). Phase transition temperature and chilling sensitivity of bovine oocytes. *Cryobiology*.

[20] Zeron Y, Pearl M, Borochov A, Arav A (1999). Kinetic and temporal factors influence chilling injury to germinal vesicle and mature bovine oocytes. *Cryobiology*.

[21] Giraud MN, Motta C, Boucher D, Grizard G (2000). Membrane fluidity predicts the outcome of cryopreservation of human spermatozoa. *Human Reproduction*.

[22] Quinn PJ (1985). A lipid-phase separation model of low-temperature damage to biological membranes. *Cryobiology*.

[23] Talaei T, Esmaeelpour T, Aekiyash F, Bahmanpour S (2010). Effects of cryopreservation on plasma membrane glycoconjugates of human spermatozoa. *Iranian Journal of Reproductive Medicine*.

[24] Benoff S (1997). Carbohydrates and fertilization: an overview. *Molecular Human Reproduction*.

[25] Cross NL, Overstreet JW (1987). Glycoconjugates of the human sperm surface: distribution and alterations that accompany capacitation in vitro. *Gamete Research*.

[26] Lassalle B, Testart J (1994). Human zona pellucida recognition associated with removal of sialic acid from human sperm surface. *Journal of Reproduction and Fertility*.

[27] O’Connell M, McClure N, Lewis SEM (2002). The effects of cryopreservation on sperm morphology, motility and mitochondrial function. *Human Reproduction*.

[28] Ford WCL, Rees JM, Gagnon C (1990). The bioenergetics of mammalian sperm motility. *Controls of Sperm Motility: Biological and Clinical Aspects*.

[29] Zamboni L (1987). The ultrastructural pathology of the spermatozoon as a cause of infertility: the role of electron microscopy in the evaluation of semen quality. *Fertility and Sterility*.

[30] Saleh RA, Agarwal A (2002). Oxidative stress and male infertility: from research bench to clinical practice. *Journal of Andrology*.

[31] Lasso JL, Noiles EE, Alvarez JG, Storey BT (1994). Mechanism of superoxide dismutase loss from human sperm cells during cryopreservation. *Journal of Andrology*.

[32] Wang X, Sharma RK, Sikka SC, Thomas AJ, Falcone T, Agarwal A (2003). Oxidative stress is associated with increased apoptosis leading to spermatozoa DNA damage in patients with male factor infertility. *Fertility and Sterility*.

[33] McClintock DS, Santore MT, Lee VY (2002). Bc1-2 family members and functional electron transport chain regulate oxygen deprivation-induced cell death. *Molecular and Cellular Biology*.

[34] Arnoult D, Gaume B, Karbowski M, Sharpe JC, Cecconi F, Youle RJ (2003). Mitochondrial release of AIF and EndoG requires caspase activation downstream of Bax/Bak-mediated permeabilization. *EMBO Journal*.

[35] Martin G, Cagnon N, Sabido O (2007). Kinetics of occurrence of some features of apoptosis during the cryopreservation process of bovine spermatozoa. *Human Reproduction*.

[36] Martinez-Soto JC, De Dioshourcade J, Gutiérrez-Adán A, Landeras JL, Gadea J (2010). Effect of genistein supplementation of thawing medium on characteristics of frozen human spermatozoa. *Asian Journal of Andrology*.

[37] Branco CS, Garcez ME, Pasqualotto FF, Erdtman B, Salvador M (2010). Resveratrol and ascorbic acid prevent DNA damage induced by cryopreservation in human semen. *Cryobiology*.

[38] Taylor K, Roberts P, Sanders K, Burton P (2009). Effect of antioxidant supplementation of cryopreservation medium on post-thaw integrity of human spermatozoa. *Reproductive BioMedicine Online*.

[39] Li Z, Lin Q, Liu R, Xiao W, Liu W (2010). Protective effects of ascorbate and catalase on human spermatozoa during cryopreservation. *Journal of Andrology*.

[40] de Paula TS, Bertolla RP, Spaine DM, Cunha MA, Schor N, Cedenho AP (2006). Effect of cryopreservation on sperm apoptotic deoxyribonucleic acid fragmentation in patients with oligozoospermia. *Fertility and Sterility*.

[41] Duru NK, Morshedi MS, Schuffner A, Oehninger S (2001). Cryopreservation-thawing of fractionated human spermatozoa is associated with membrane phosphatidylserine externalization and not DNA fragmentation. *Journal of Andrology*.

[42] Isachenko E, Isachenko V, Katkov II (2004). DNA integrity and motility of human spermatozoa after standard slow freezing versus cryoprotectant-free vitrification. *Human Reproduction*.

[43] Petyim S, Choavaratana R (2006). Cryodamage on sperm chromatin according to different freezing methods, assessed by AO test. *Journal of the Medical Association of Thailand*.

[44] Spanò M, Cordelli E, Leter G, Lombardo F, Lenzi A, Gandini L (1999). Nuclear chromatin variations in human spermatozoa undergoing swim-up and cryopreservation evaluated by the flow cytometric sperm chromatin structure assay. *Molecular Human Reproduction*.

[45] Zribi N, Feki Chakroun N, El Euch H, Gargouri J, Bahloul A, Ammar Keskes L (2010). Effects of cryopreservation on human sperm deoxyribonucleic acid integrity. *Fertility and Sterility*.

[46] Thomson LK, Fleming SD, Aitken RJ, De Iuliis GN, Zieschang JA, Clark AM (2009). Cryopreservation-induced human sperm DNA damage is predominantly mediated by oxidative stress rather than apoptosis. *Human Reproduction*.

[47] Evenson DP, Darzynkiewicz Z, Melamed MR (1980). Comparison of human and mouse sperm chromatin structure by flow cytometry. *Chromosoma*.

[48] Evenson DP, Melamed MR (1983). Rapid analysis of normal and abnormal cell types in human semen and testis biopsies by flow cytometry. *Journal of Histochemistry and Cytochemistry*.

[49] Kalthur G, Adiga SK, Upadhya D, Rao S, Kumar P (2008). Effect of cryopreservation on sperm DNA integrity in patients with teratospermia. *Fertility and Sterility*.

[50] Paasch U, Sharma RK, Gupta AK (2004). Cryopreservation and thawing is associated with varying extent of activation of apoptotic machinery in subsets of ejaculated human spermatozoa. *Biology of Reproduction*.

[51] Aitken RJ, De Iuliis GN (2007). Origins and consequences of DNA damage in male germ cells. *Reproductive BioMedicine Online*.

[52] Tarozzi N, Bizzaro D, Flamigni C, Borini A (2007). Clinical relevance of sperm DNA damage in assisted reproduction. *Reproductive BioMedicine Online*.

[53] Zini A, Boman JM, Belzile E, Ciampi A (2008). Sperm DNA damage is associated with an increased risk of pregnancy loss after IVF and ICSI: systematic review and meta-analysis. *Human Reproduction*.

[54] Cross NL, Hanks SE (1991). Effects of cryopreservation on human sperm acrosomes. *Human Reproduction*.

[55] Royere D, Hamamah S, Nicolle JC, Lansac J (1991). Chromatin alterations induced by freeze-thawing influence the fertilizing ability of human sperm. *International Journal of Andrology*.

[56] Sakkas D, Urner F, Bianchi PG (1996). Sperm chromatin anomalies can influence decondensation after intracytoplasmic sperm injection. *Human Reproduction*.

[57] Friedler S, Strassburger D, Raziel A, Komarovsky D, Soffer Y, Ron-El R (1997). Intracytoplasmic injection of fresh and cryopreserved testicular spermatozoa in patients with nonobstructive azoospermia—A comparative study. *Fertility and Sterility*.

[58] Ben Rhouma K, Marrakchi H, Khouja H, Attalah K, Ben Miled E, Sakly M (2003). Outcome of intracytoplasmic injection of fresh and frozen-thawed testicular spermatozoa: a comparative study. *Journal of Reproductive Medicine for the Obstetrician and Gynecologist*.

[59] Habermann H, Seo R, Cieslak J, Niederberger C, Prins GS, Ross L (2000). In vitro fertilization outcomes after intracytoplasmic sperm injection with fresh or frozen-thawed testicular spermatozoa. *Fertility and Sterility*.

[60] Huang FJ, Chang SY, Tsai MY (2000). Clinical implications of intracytoplasmic sperm injection using cryopreserved testicular spermatozoa from men with azoospermia. *Journal of Reproductive Medicine for the Obstetrician and Gynecologist*.

[61] Croo ID, Van Der Elst J, Everaert K, Sutter PD, Dhont M (1998). Fertilization, pregnancy and embryo implantation rates after ICSI with fresh or frozen-thawed testicular spermatozoa. *Human Reproduction*.

[62] Tournaye H, Merdad T, Silber S (1999). No differences in outcome after intracytoplasmic sperm injection with fresh or with frozen-thawed epididymal spermatozoa. *Human Reproduction*.

[63] Sukcharoen N, Sithipravej T, Promviengchai S, Chinpilas V, Boonkasemsanti W (2001). Comparison of the outcome of intracytoplasmic sperm injection using fresh and frozen-thawed epididymal spermatozoa obtained by percutaneous epididymal sperm aspiration. *Journal of the Medical Association of Thailand*.

[64] Cayan S, Lee D, Conaghan J (2001). A comparison of ICSI outcomes with fresh and cryopreserved epididymal spermatozoa from the same couples. *Human Reproduction*.

[65] Shibahara H, Hamada Y, Hasegawa A (1999). Correlation between the motility of frozen-thawed epididymal spermatozoa and the outcome of intracytoplasmic sperm injection. *International Journal of Andrology*.

[66] Kuczyski W, Dhont M, Grygoruk C, Grochowski D, Woczyski S, Szamatowicz M (2001). The outcome of intracytoplasmic injection of fresh and cryopreserved ejaculated spermatozoa—A prospective randomized study. *Human Reproduction*.

[67] Ragni G, Caccamo AM, Dalla Serra A, Guercilena S (1990). Computerized slow-staged freezing of semen from men with testicular tumors or Hodgkin’s disease preserves sperm better than standard vapor freezing. *Fertility and Sterility*.

[68] Borges E, Rossi LM, Locambo de Freitas CV (2007). Fertilization and pregnancy outcome after intracytoplasmic injection with fresh or cryopreserved ejaculated spermatozoa. *Fertility and Sterility*.

[69] Borini A, Sereni E, Bonu MA, Flamigni C (2000). Freezing a few testicular spermatozoa retrieved by TESA. *Molecular and Cellular Endocrinology*.

[70] Cohen J, Garrisi GJ, Congedo-Ferrara TA, Kieck KA, Sehimmel TW, Scott RT (1997). Cryopreservation of single human spermatozoa. *Human Reproduction*.

[71] Walmsley R, Cohen J, Ferrara-Congedo T, Reing A, Garrisi J (1998). The first births and ongoing pregnancies associated with sperm cryopreservation within evacuated egg zonae. *Human Reproduction*.

[72] Montag M, Rink K, Dieckmann U, Delacrétaz G, Van Der Ven H (1999). Laser-assisted cryopreservation of single human spermatozoa in cell- free zona pellucida. *Andrologia*.

[73] Hsieh YY, Tsai HD, Chang CC, Lo HY (2000). Cryopreservation of human spermatozoa within human or mouse empty zona pellucidae. *Fertility and Sterility*.

[74] Liu J, Zheng XZ, Baramki TA, Compton G, Yazigi RA, Katz E (2000). Cryopreservation of a small number of fresh human testicular spermatozoa and testicular spermatozoa cultured in vitro for 3 days in an empty zona pellucida. *Journal of Andrology*.

[75] Levi-Setti PE, Albani E, Negri L, Cesana A, Novara P, Bianchi S (2003). Cryopreservation of a small number of spermatozoa in yolk-filled human zonae pellucidae. *Archivio Italiano di Urologia e Andrologia*.

[76] Cesana A, Novara P, Bianchi S Sperm cryopreservation in oligo-asthenospermic patients.

[77] Hassa H, Gurer F, Yildirim A, Can C, Sahinturk V, Tekin B (2006). A new protection solution for freezing small numbers of sperm inside empty zona pellucida: Osmangazi-Turk solution. *Cell Preservation Technology*.

[78] Gil-Salom M, Romero J, Rubio C, Ruiz A, Remohí J, Pellicer A (2000). Intracytoplasmic sperm injection with cryopreserved testicular spermatozoa. *Molecular and Cellular Endocrinology*.

[79] Sereni E, Bonu MA, Fava L (2008). Freezing spermatozoa obtained by testicular fine needle aspiration: a new technique. *Reproductive BioMedicine Online*.

[80] Quintans C, Donaldson M, Asprea I (2000). Development of a novel approach for cryopreservation of very small numbers of spermatozoa. *Human Reproduction*.

[81] Bouamama N, Briot P, Testart J (2003). Comparison of two methods of cryoconservation of sperm when in very small numbers. *Gynecologie Obstetrique Fertilite*.

[82] Gvakharia M, Adamson G (2001). A method of successful cryopreservation of small numbers of human spermatozoa. *Fertility and Sterility*.

[83] Sohn JO, Jun SH, Park LS (2003). Comparison of recovery and viability of sperm in ICSI pipette after ultra rapid freezing or slow freezing. *Fertility and Sterility*.

[84] Just A, Gruber I, Wöber M, Lahodny J, Obruca A, Strohmer H (2004). Novel method for the cryopreservation of testicular sperm and ejaculated spermatozoa from patients with severe oligospermia: a pilot study. *Fertility and Sterility*.

[85] Herrler A, Eisner S, Bach V, Weissenborn U, Beier HM (2006). Cryopreservation of spermatozoa in alginic acid capsules. *Fertility and Sterility*.

[86] Nawroth F, Isachenko V, Dessole S (2002). Vitrification of human spermatozoa without cryoprotectants. *Cryo-Letters*.

[87] Schuster TG, Keller LM, Dunn RL, Ohl DA, Smith GD (2003). Ultra-rapid freezing of very low numbers of sperm using cryoloops. *Human Reproduction*.

[88] Desai N, Culler C, Goldfarb J (2004). Cryopreservation of single sperm from epidydimal and testicular samples on cryoloops: preliminary case report. *Fertility and Sterility*.

[89] Desai NN, Blackmon H, Goldfarb J (2004). Single sperm cryopreservation on cryoloops: an alternative to hamster zona for freezing individual spermatozoa. *Reproductive BioMedicine Online*.

[90] Isaev DA, Zaletov SY, Zaeva W Artificial microcontainers for cryopreservation of solitary spermatozoa.

[91] Desai N, Glavan D, Goldfarb J (1998). A convenient technique for cryopreservation of micro quantities of sperm. *Fertility and Sterility*.

[92] Isachenko V, Isachenko E, Montag M (2005). Clean technique for cryoprotectant-free vitrification of human spermatozoa. *Reproductive BioMedicine Online*.

[93] Koscinski I, Wittemer C, Lefebvre-Khalil V, Marcelli F, Defossez A, Rigot JM (2007). Optimal management of extreme oligozoospermia by an appropriate cryopreservation programme. *Human Reproduction*.

[94] Hamamah S, Royere D, Nicolle JC, Paquignon M, Lansac J (1990). Effects of freezing-thawing on the spermatozoon nucleus: a comparative chromatin cytophotometric study in the porcine and human species. *Reproduction Nutrition Development*.

[95] Hammadeh ME, Dehn C, Hippach M (2001). Comparison between computerized slow-stage and static liquid nitrogen vapour freezing methods with respect to the deleterious effect on chromatin and morphology of spermatozoa from fertile and subfertile men. *International Journal of Andrology*.

[96] Gandini L, Lombardo F, Lenzi A, Spanò M, Dondero F (2006). Cryopreservation and sperm DNA integrity. *Cell and Tissue Banking*.

[97] Ngamwuttiwong T, Kunathikom S (2007). Evaluation of cryoinjury of sperm chromatin according to liquid nitrogen vapour method (I). *Journal of the Medical Association of Thailand*.

[98] Dejarkom S, Kunathikom S (2007). Evaluation of cryo-injury of sperm chromatin according to computer controlled rate freezing method part 2. *Journal of the Medical Association of Thailand*.

[99] Ahmad L, Jalali S, Shami SA, Akram Z, Batool S, Kalsoom O (2010). Effects of cryopreservation on sperm DNA integrity in normospermic and four categories of infertile males. *Systems Biology in Reproductive Medicine*.

[100] Høst E, Lindenberg S, Kahn JA, Christensen F (1999). DNA strand breaks in human sperm cells: a comparison between men with normal and oligozoospermic sperm samples. *Acta Obstetricia et Gynecologica Scandinavica*.

[101] Steele EK, McClure N, Lewis SEM (2000). Comparison of the effects of two methods of cryopreservation on testicular sperm DNA. *Fertility and Sterility*.

